# Metal [100] Nanowires with Negative Poisson’s Ratio

**DOI:** 10.1038/srep27560

**Published:** 2016-06-10

**Authors:** Duc Tam Ho, Soon-Yong Kwon, Sung Youb Kim

**Affiliations:** 1 Department of Mechanical Engineering, Ulsan National Institute of Science and Technology, Ulsan 44919, South Korea; 2 School of Materials Science and Engineering, Ulsan National Institute of Science and Technology, Ulsan 44919, South Korea

## Abstract

When materials are under stretching, occurrence of lateral contraction of materials is commonly observed. This is because Poisson’s ratio, the quantity describes the relationship between a lateral strain and applied strain, is positive for nearly all materials. There are some reported structures and materials having negative Poisson’s ratio. However, most of them are at macroscale, and reentrant structures and rigid rotating units are the main mechanisms for their negative Poisson’s ratio behavior. Here, with numerical and theoretical evidence, we show that metal [100] nanowires with asymmetric cross-sections such as rectangle or ellipse can exhibit negative Poisson’s ratio behavior. Furthermore, the negative Poisson’s ratio behavior can be further improved by introducing a hole inside the asymmetric nanowires. We show that the surface effect inducing the asymmetric stresses inside the nanowires is a main origin of the superior property.

Materials with a negative Poisson’s ratio (auxetic materials) expand rather than contract along a lateral direction when they are subjected to stretch. Auxetic materials have attracted considerable attention due to their great potential applications such as textile fabrics^1^, and the aerospace industry^2^. Some crystal structures^3^,^4^,^5^,^6^, models of materials^7^,^8^, and some materials at volume-phase transition^9^ can show the auxetic behavior. However, the main mechanisms for auxeticity were reentrant structures and rotating rigid units, and many reported materials were at bulk scale^10^,^11^,^12^,^13^. There were several efforts to discover and tailor nanoscale materials with auxetic behavior. For example, the discovery of auxeticity in single layer black phosphorous^14^, and the tailoring of graphene with defects to show auxeticity^15^. Our previous studies^16^,^17^ revealed that metal (001) nanoplates can also show negative Poisson’s ratio even though their bulk counterparts exhibit positive Poisson’s ratio.

One-dimensional nanoscale materials including metal nanowires and metal nanotubes have been become an attractive research topic due to their remarkable material properties including mechanical properties such as large elastic range^18^, high ideal strength compared to their bulk counterparts^19^. Some fabrication methods have been reported to synthesize metal nanowires and nanotubes with size even a few atomic layers^20^,^21^. There are extensive studies have been conducted to understand mechanical properties of the metal one-dimensional nanoscale materials by theoretical, computational, and experimental approaches^22^,^23^,^24^. Origin of the unusual mechanical properties in the nanoscale materials mainly comes from their large surface-to-volume ratio and large surface stress^19^,^24^,^25^,^26^. Large surface stress is a main reason for the auxeticity in metal (001) nanoplates^16^. At bulk-scale, when the material is under uniaxial loading, there is a sudden contraction and expansion (branching) of crystal structure along lateral directions at a critical strain. However, at nanoscale, due to the effect of surface stress, the sudden contraction and expansion are replaced by a gradual contraction along in-plane lateral direction and expansion along the thickness direction. As the result, there is a negative Poisson’s ratio along the out-of-plane direction of the metal (001) nanoplates.

In this study, we show that one-dimensional nanoscale materials can show auxeticity with proper designs. We investigate the effect of asymmetry degree of cross-section of nanowires on their Poisson’s ratios through atomistic simulations. Our simulation results demonstrate that Poisson’s ratios of the nanowires can be effectively governed by the aspect ratio of the cross-section. For symmetric cross-section, i.e., unity aspect ratio, the nanowires show positive Poisson’s ratio as usual expectation. However, as the aspect ratio increases, a Poisson’s ratio component decreases, and it even becomes negative at finite strain. Besides, we show that the auxeticity of nanowires can be further improved by introducing a hole inside the nanowires. It is found that the surface relaxation that generates asymmetric stresses inside nanowires is a main origin of the auxetic behavior of the metal nanowires.

## Negative Poisson’s ratio in [100] rectangular nanowires

**Figure 1:**
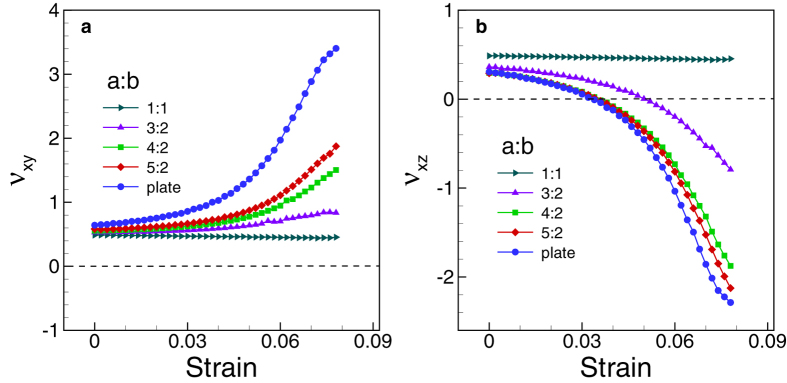
Poisson’s ratios of Au nanowires with different aspect ratios under loading. (**a**) The Poisson’s ratio component *ν*_*xy*_ and (**b**) The Poisson’s ratio component *ν*_*xz*_. All nanostructures have the same thickness of 10a_0_. Poisson’s ratios the SNW (*a* = *b*) are positive and nearly constant, while those of the RNWs (*a* > *b*) change drastically under loading. When the aspect ratio is 2 (4:2), the Poisson’s ratio component *ν*_*xz*_ of the RNW is close to that of the nanoplate.

We employ molecular statics (MS) simulation to predict mechanical response of nanoscale materials. For convenience of notation, we assign here the *x*-, *y*-, and *z*-directions to the [100]-, [010]-, and [001]-directions, respectively. More details on the simulation technique can be seen in the Simulation Methods. We firstly present Poisson’s ratio components *ν*_*xy*_ and *ν*_*xz*_ of an Au (001) nanoplate and Au (001) nanowires with the cross-sectional area of *a* × *b* where *a* is the width along the *y*-direction and *b* is the thickness along the *z*-direction in Fig. 1. *b* is kept as 10a_0_ where a_0_ is the lattice parameter while *a* is various with the ratio *r* = *a/b* = 1.0, 1.5, 2.0, 2.5, and ∞. The interaction between the Au atoms here is described by the embedded-atom-method (EAM) potential model developed by Foiles *et al*^27^. When *r* = 1.0, the cross-sectional shape is square, ν_xy_ = *ν*_*xz*_ = *ν* = 0.49 at the unstrained state, and it seems not to change with applied strain. Poisson’s ratio component of the square nanowire (SNW) is larger than that of the bulk counterpart (0.46) due to surface effect. Detail of surface effect on Poisson’s ratio of SNWs can be seen in the work by Dingreville *et al*^22^. As *r* = ∞ (nanoplate), we can see that the nanoplate has two distinct Poisson’s ratios, and they show strong dependence on applied strain. The component *ν*_*xy*_ starts from 0.64 at the unstrained state and then it increases. On the other hand, *ν*_*xz*_ is 0.31 at zero strain, decreases with increasing of applied strain, and reaches a negative value at a strain of 0.034. The strain at which materials shows negative Poisson’s ratio is called critical auxetic strain. When *r* is larger than 1.0 (but still a nanowire), it is interesting that a negative Poisson’s ratio is still observed (Fig. 1b). Poisson’s ratio behavior is dependent on the aspect ratio *r* and it approaches to that of the nanoplate as *r* increases. It is noteworthy to mention that as the aspect ratio is 2.0, the difference of the components *ν*_*xz*_ between the nanoplate and that of the nanowires is small. For example, at unstrained state, *ν*_*xz*_ of the nanowire is approximately 0.30, and it decreases with increase of the strain as well. In addition, the critical auxetic strain of the nanowire is also the same as that of the nanoplate. This is the first time metal nanowires are found to show auxeticity.

## Effect of surface stress on Poisson’s ratio of [100] rectangular nanowires

**Figure 2:**
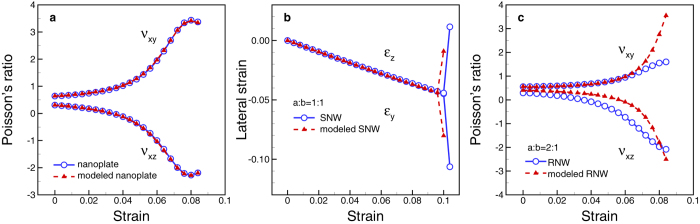
Comparison of mechanical responses of Au nanoplate, SNW, and RNW with their corresponding bulk models. (**a**) nanoplate versus modeled nanoplate. (**b**) SNW versus modeled SNW, and (**c**) RNW versus modeled RNW. All nanoscale structures have the same thickness of 10a_0_. The mechanical responses of the modeled SNW and modeled nanoplate are in good agreement with the SNW and nanoplate, respectively. In the case of the RNW, although the Poisson’s ratio *ν*_*xz*_ of the RNW is relatively different from that of the modeled RNW, the overall mechanical responses of the two structures are similar.

When a material is under uniaxial loading along the *x*-direction, only one stress component *σ*_*x*_ is non-zero, and the other five components are zero. For bulk material, local stress at any point in its domain also follows this condition. However, for a nanoscale material under the same loading condition, stress at a point in its domain is not necessary to have a single non-zero stress component. Rather, due to large tensile stress at free surfaces, stress in atoms in the interior part of the nanoscale material is compressive. Here, interior part means all atoms of the nanoscale material except atoms on several layers from each free surface. The compressive stress along the in-plane lateral direction inside a (001) nanoplate induced by tensile surface stress is found to be inversely proportional to its thickness: 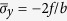 where *f* is the surface stress^16^. Details on the mechanism of the induced compressive stresses inside nanostructures can be found in the works of Diao *et al*^28^,^29^. To understand the mechanical behavior of nanoscale materials, it is very useful to introduce their corresponding bulk counterparts. For example, the mechanical behavior of a nanoplate under the uniaxial loading condition is approximately equivalent to that of the bulk counterpart under multiaxial loading condition in which tensile loading is applied along the *x*-direction, and a finite stress 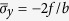 is applied along the *y*-direction^16^. We name the bulk counterpart under this loading condition modeled nanoplate in Fig. 2a. The compressive stress, which is automatically induced by the surface stress in case of metal nanoplates, dilutes the sudden branching of crystal structure and thus makes the negative Poisson’s ratio^16^. However, when we consider a SNW, we never see the auxetic behavior (Fig. 1b). With increasing of applied strain, SNWs deform gradually with positive Poisson’s ratio behavior, and it then may fail with a phase transformation or other mechanisms such as slip, twinning etc^30^. In the case of a SNW with the width of *b*, the compressive stresses induced by the surface stress are 
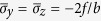
. Now, the mechanical behavior of a SNW under the uniaxial stress condition is approximately equivalent to that of the bulk counterpart under multiaxial loading condition in which tensile loading is applied along the *x*-direction and the same amount of stresses 
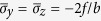
 are applied along the *y*- and *z*-directions, which we name modeled SNW (Fig. 2b). As shown in Fig. 2b, under these symmetric transverse stresses, the lateral strains of the modeled SNW always decrease with increasing of applied strain until a phase transformation takes place. This confirms that the modeled SNW always exhibits a positive Poisson’s ratio before it becomes unstable. It is noteworthy that the mechanical responses of a SNW and its model are in good agreement (Fig. 2b). On the other hand, when we consider a rectangular nanowire (RNW) with the cross-section *a* × *b* (supposing *a* > *b*), the induced stresses are approximately 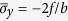 and 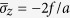 along the *y*- and *z*-directions, respectively. Note that as *a* approaches to infinite, there is no induced stress along the *z*-direction. Now, the RNW can be regarded as the bulk crystal under multiaxial loading which is a combination of a tensile loading along the *x*-direction, an applied stress along the *y*-direction 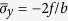, and an applied stress along the *z*-direction 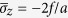. The induced stresses along the lateral directions in RNWs are asymmetric. The corresponding bulk crystal under this loading condition is named as modeled RNW. As shown in Fig. 2c, although the change in the Poisson’s ratios of the modeled RNW and those of the RNW are relatively different from each other in term of numbers, the overall tendencies of the two structures are similar, i.e., the Poisson’s ratio along the z-direction becomes negative at finite strain, whereas the Poisson’s ratio along the y-direction is always positive. The difference in term of numbers, which originates from tensile stress zone in RNWs, will be discussed in the next paragraph. From the observation of metal nanoplates, SNWs, and RNWs, we may conclude that the auxetic behavior of RNWs (as well as nanoplate) originates from the asymmetry of the stresses in the interior part in the lateral directions which are intrinsically induced by the surface relaxation of RNWs. The degree of asymmetry of the induced stresses depends on the aspect ratio *r* of the cross-section of RNWs. In general, the more asymmetric induced stresses generate, the larger negative Poisson’s ratio, as shown in Fig. 1b. If there is no asymmetry (*r* = 1.0), then the Poisson’s ratios of nanowires are always positive. We will discuss later that this mechanism is the unique characteristic of cubic materials under loading along [100]-direction.

As mentioned early, when the aspect ratio is approximately 2.0, the change of the Poisson’s ratio component *ν*_*xz*_ of a RNW is close to that of the corresponding nanoplate. To understand this behavior, we compare the distributions of the induced stresses in the cross-section of the SNW, RNW, and nanoplate in Figure S1. For all cases, the induced stresses at the interior part of the nanostructures are compressive and relatively homogeneous, except the stress component *σ*_*z*_ of the RNW with the aspect ratio 2.0. The stress is not homogeneous and it is more tensile at the center of the RNW. We further investigate the stress distribution *σ*_*z*_ of RNWs with different aspect ratios in Figure S2. Remarkably, for RNWs with the aspect ratio larger than 1.6, there always exists a tensile stress zone at which the stress *σ*_*z*_ is even tensile. Furthermore, as the aspect ratio increases (>3.0), it is split into two tensile stress zones that are positioned at the same distance with the thickness from each side surface, as shown in Figures S2 and S3. Further detail of the tensile stress zones inside the RNWs is discussed in the Supplementary Information. It is noteworthy that the degree of asymmetry of the induced stresses at the tensile stress zones are larger that of the average induced stresses. As shown in Fig. 2c, Poisson’s ratio along the z-direction of the modeled RNW is less auxetic than that of the RNW. This is because the model reflects only the average induced stresses, and thus it does not consider the high degree of asymmetry of the induced stresses in the tensile stress zones. Consequently, the auxeticity of the RNW becomes larger, and the Poisson’s ratio along the z-direction of a RNW with the aspect ratio approximately 2.0 is close to that of the corresponding nanoplate. It is noting that the modeled SNW and modeled nanoplate can excellently capture the mechanical responses of the SNW and nanoplate (Fig. 2a,b) because these nanostructures do not have the tensile stress zones. The tensile stress zones that enhance the auxeticity of the RNWs are unique and intrinsic characteristics of the RNWs with large aspect ratios.

## Rectangular Nanotubes

**Figure 3:**
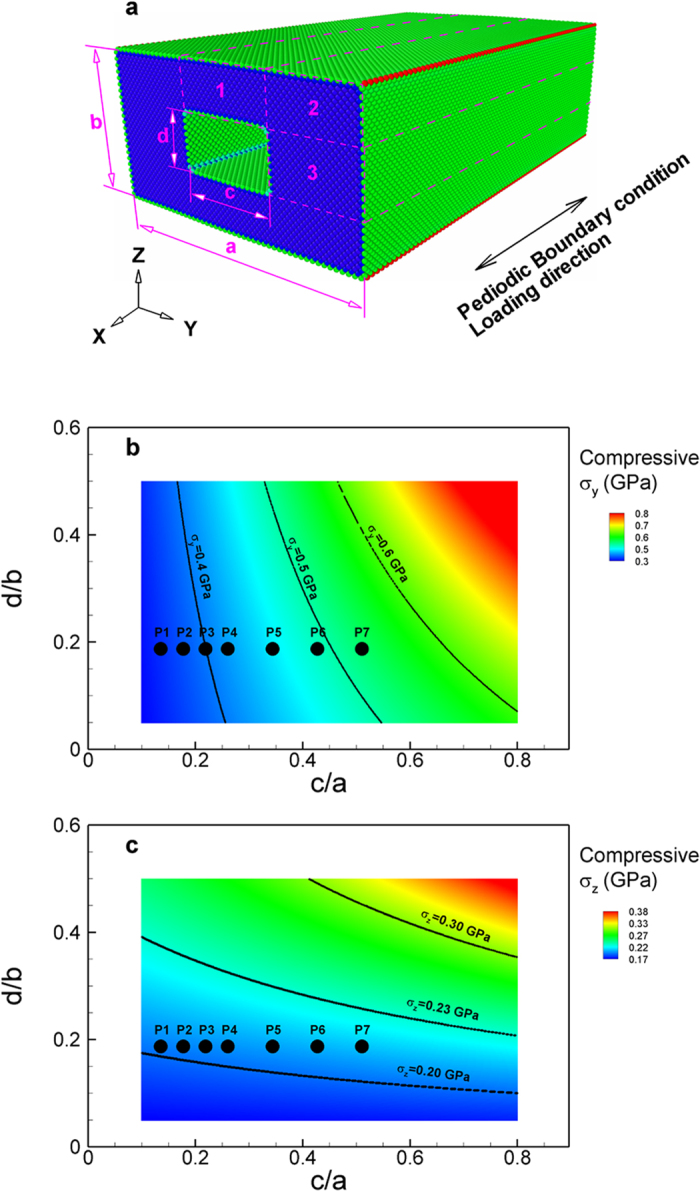
A RNT model and the landscape of the average induced stresses in interior part of the RNW with different sizes of a hole. (**a**) A RNT model, (**b**) The average induced stress along the y-direction 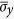, and (**c**) The average induced stress along the z-direction 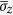. The RNTs have the fixed values of *a* = 48a_0_, and *b* = 24a_0_. The induced stresses are strongly dependent on the dimension of the hole.

So far, we have shown that, owing to surface relaxation there are induced compressive stresses along the lateral directions inside RNWs and that the asymmetry of the induced stresses is the main origin of the auxetic behavior of the nanowires at finite strain. Since induced stress is proportional to surface stress and the inverse of the size, the auxeticity of RNWs can be tuned by adjusting surface stress and geometry. For example, in order to enhance auxeticity, one may increase the aspect ratio *r* = *a/b* of RNWs (Fig. 1b) so that the asymmetry of cross-section increases. Selecting a material having larger surface stress so that the surface stress induces larger compressive stresses inside the RNWs is also a possible way. This issue will be discussed later. In this section, we introduce another way to enrich surface effect on the overall mechanical property of RNWs. In particular, a hole is introduced by deleting a volume (*c* × *d* × *L*) at the center of a RNW as shown in Fig. 3a. Now, the nanowire becomes a rectangular hollow nanowire or rectangular nanotube (RNT). Metal nanotubes have been become an attractive research recently^31^,^32^,^33^,^34^. Here, metal RNTs with the hole inside have larger surface-to-volume ratio than that of the corresponding RNWs. This larger surface-to-volume ratio as well as geometric asymmetry can provoke larger asymmetry degree of the induced stresses, and, therefore the auxeticity of the nanostructures can be enhanced.

In order to design larger auxetic metal RNTs, the stresses induced by free surfaces inside the RNTs should be understood in advance. Figure 3a presents the model of a RNT in which the solid part of the RNT can be divided into three kinds of region according to the influence by different surfaces. Due to the tensile stress at free surfaces, there are also compressive stresses in atoms in the interior part of the structure. The average stresses in the entire RNT along the *y*-direction 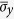 and the *z*-direction 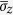 can be given as:




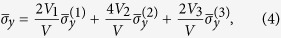







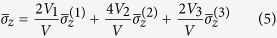




where *V*_*1*_, *V*_*2*_, and *V*_*3*_ are the volumes of regions (1), (2), and (3) (presented in Fig. 3a), respectively and 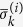 is the average stress in the *k*-direction of region (*i*). The average stresses of each region can be approximated as:




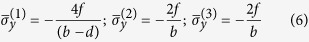







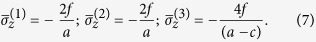




Substituting Eqs (6) and (7) to Eqs (4) and (5), respectively, we obtain:




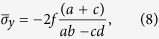







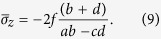




**Figure 4:**
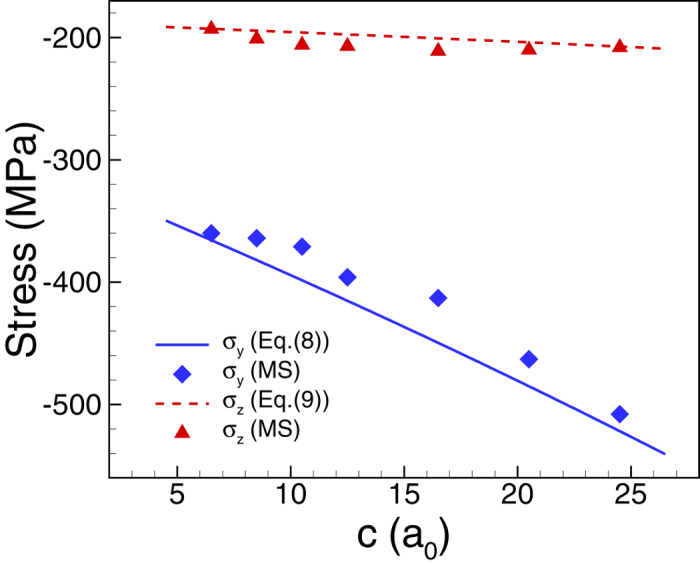
The comparison of the stress calculation by Eqs (8) and (9) with the direct MS calculation. The results by the two methods are in good agreement.

Clearly, the average stresses 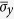 and 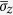 are dependent on the surface stress and four geometric parameters a, *b*, *c*, and *d*. Note that if *c* = 0 then *d* should be vanished and vice versa. Figure 3b,c present the changes of the magnitudes of the compressive stresses 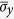 and 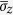 with the changing of the parameters *c* and *d*, respectively, when the parameters *a* and *b* are fixed as *a* = 48a_0_ and *b* = 24a_0_. While 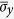 is more sensitive to *c*, 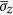 shows strongly dependent on *d*. For the case of 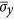, the slopes of the contour lines are relatively large; especially the contour lines are nearly parallel to the vertical axis with small values of *c*. On the other hand, slopes of the contour lines of 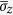 are relatively small and the contour lines seem to be normal to the vertical axis with small value of *d*. To confirm the stress calculation before designing RNTs with large auxetic, we compare the stresses obtained by Eqs (8) and (9) with the stresses directly calculated from MS simulation. In the MS simulation, we calculate the average stresses 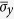 and 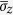 of different RNTs with various values of *c* and *d* = 4a_0_ that are marked as P_1_ to P_7_ in Fig. 3b,c. 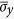 and 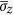 are calculated by averaging stress of all atoms in the RNWs except three outmost layers from free surfaces. As shown in Fig. 4, the predictions of both Eqs (8) and (9) are in good agreement with the results obtained by MS calculation.

**Figure 5:**
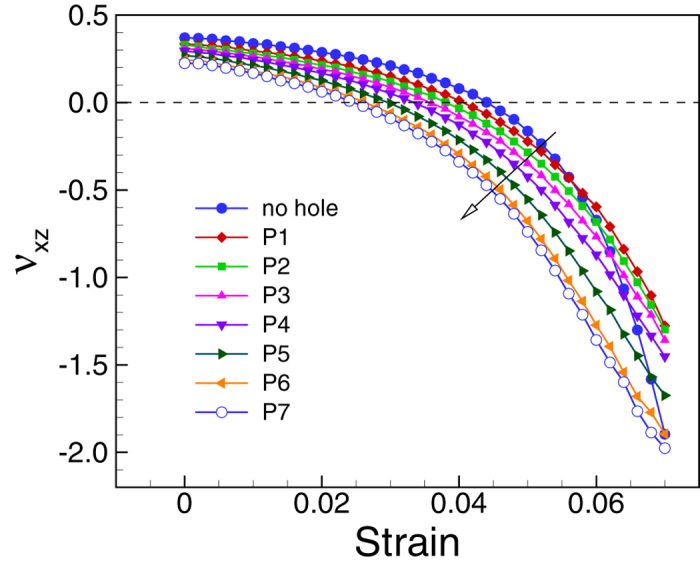
Effect of the size of the hole on Poisson’s ratios of Au RNWs. Different sizes of the hole, that are marked as P_1_ to P_7_ in Fig. 3b,c, are introduced at the center of the same RNWs with *a* = 48a_0_, and *b* = 24a_0_. It is clear that the Poisson’s ratio of the RNTs can be effectively controlled by changing the size of hole.

Now, we investigate the effect of the dimensions of the hole on the auxeticity of the RNTs. Again, the key idea here to enhance the auxeticity for RNT is selecting the parameters *c* and *d* such that the degrees of asymmetry of the induced stresses as well as the values of the induced stresses are large as much as possible. Based on the changes of the induced stresses with the parameters *c* and *d* shown in Fig. 3b,c, a RNT can exhibit larger auxetic with large values of *c* and small values of *d.* To illustrate the design, we calculate Poisson’s ratio of RNTs with dimensions the same as mentioned above, i.e., *a*, *b*, and *d* are fixed as *a* = 48a_0_, *b* = 24a_0_, and *d* = 4a_0_ and different values of *c* that are marked as P_1_ to P_7_ in Fig. 3b,c. As shown in Fig. 4, the compressive value of 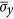 linearly increases with the increase of *c*, whereas the compressive value of 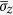 seems relatively unchanged with *c*. Thus, the asymmetry degree of the induced stresses increases as the size of the hole varies from P_1_ to P_7_. We then may expect that the auxeticity of the RNTs will increase with increasing of *c.* Fig. 5 shows the changes of the Poisson’s ratio component *ν*_*xz*_ of the different RNTs with applied strain. The Poisson’s ratio is strongly dependent on the parameters *c.* For all considered cases, the RNTs are more auxetic than the corresponding RNW (as *c* = *d* = 0). As we expected, the auxeticity of the RNTs consistently increases with the change of the dimension from P_1_ to P_7_. And, with a proper selection of the value of *c*, the auxeticity can be significantly improved. For example, the RNW has the auxetic strain of 0.044, whereas the RNT with *c* = 24a_0_ nm and d = 4a_0_ shows auxetic behavior at a strain of 0.025. It is worth noting that the RNTs are also more auxetic than the nanoplate with the same thickness. Therefore, the auxeticity can be significantly improved by the designing.

## Negative Poisson’s ratio in other nanowires

**Figure 6:**
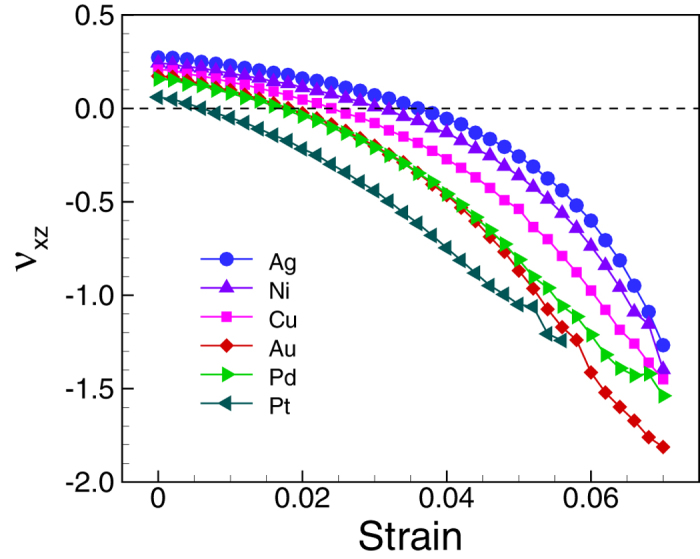
Poisson’s ratio of different metal RNTs. For all metals, the RNTs have the same size (*a* = 36a_0_, *b* = 18a_0_, *c* = 12a_0_, and *d* = 2a_0_). Poisson’s ratios are significantly different from each other at nanoscale, even though they are almost the same at bulk-scale. The auxeticity increases in the following order: Ag, Ni, Cu, Au, Pd, and Pt, which is exactly the same order in which the surface stresses of the employed metals increase.

Auxeticity can also be found in other metals. We compare Poisson’s ratio behaviors of six RNTs of the following six metals: Cu, Ag, Au, Ni, Pd, and Pt. The RNTs are assumed to have the same size of which *a* = 36*a*_*0*_, *b* = 18*a*_*0*_, *c* = 12*a*_*0*_ and *d=*2*a*_*0*_. In Table 1, we list the Poisson’s ratios of the RNTs as well as their corresponding bulk metals at unstrained states. Poisson’s ratios of these metals at bulk-scale are almost the same and they are in the range of 0.41 (Ni) and 0.47 (Pt). However, in the case of the RNTs, the Poisson’s ratios of the metallic structures are different from each other although they have the same geometry. Among the considered RNTs, *ν*_*xz*_ of the Pt RNT is smallest (almost zero) while that of the Ag RNT is the largest (0.27). The Poisson’s ratio component *ν*_*xz*_ of each RNT is smaller than that of the corresponding bulk metal. As presented in Table 1, the magnitude of the decrease is in the following order: Pt, Pd, Au, Cu, Ni, and Ag. Furthermore, as showed in Fig. 6, as stretch increases, the Poisson’s ratio component *ν*_*xz*_ of all the metal RNTs decrease more, and again the magnitude of decreases are different from each other. The critical auxetic strain of the Pt RNT is the smallest (0.007) and that of the Ag nanotube is the largest (0.036). Similarly, at certain strain, the Pt RNT is the most auxetic and the Ag nanotube is the least auxetic. The degree of auxeticity of the metals is in the following order (from the highest to the lowest): Pt, Pd, Au, Cu, Ni, and Ag. This phenomenon was also observed in the case of metal (001) nanoplates^17^.

We have shown that Poisson’s ratios of the RNTs with different base metals are largely different from each other’s not only at unstrained state but also at finite strain. It is interesting because Poisson’s ratios of the bulk metals are almost the same and all of the RNTs have the same geometry. Therefore, the base metal is also an important factor in Poisson’s ratio behavior of the nanostructures. In Table 1, we list the surface stress along [100]-direction in (001) plane of the metals. Remarkably, the magnitude of the surface stresses has the same order with the order of the degree of auxeticity above. With the same geometry, the induced compressive stresses inside the nanoscale metals are larger with larger surface stress, resulting in higher degree of auxeticity. It indicates the importance of surface stress on Poisson’s ratio of the nanostructures. Therefore, simply by changing the base metal, we can change drastically the auxeticity of metal nanowires and nanotubes.

## Discussion

**Figure 7:**
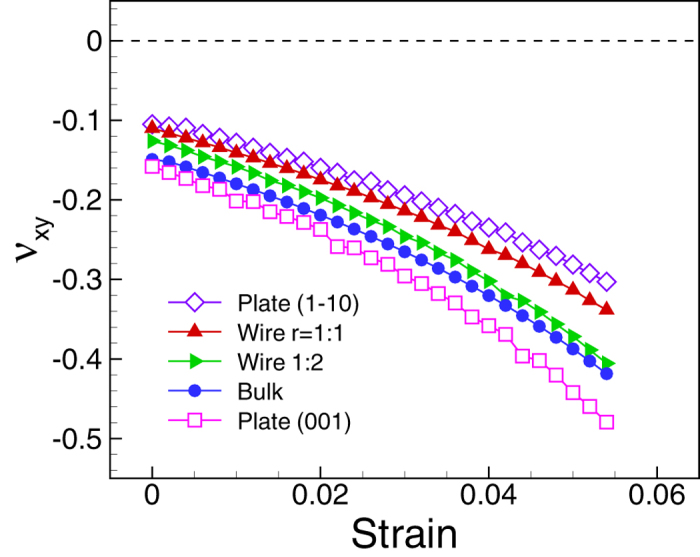
Poisson’s ratio component of Au [110]-nanowires and that of the corresponding bulk. The [110]-, 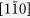-, and [001]-directions are assigned to be x-, y-, and z- directions, respectively. The dimension of the nanowires and the nanoplate along the z-direction is 17a_0_. It is clear that Au shows negative Poisson’s ratio along the 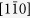-direction when it is stretched along the [110]-direction regardless of size.

We have shown that geometry of the cross-section and surface stress are the two origins for negative Poisson’s ratio behavior of metal [100] RNWs and RNTs. It is natural to ask whether the nanostructures with different crystalline orientations show the special behavior. The answer is in the following. If a crystalline solid at bulk-scale has negative Poisson’s ratio when it is stretched along a direction, it might be possible to observe auxeticity at nanoscale. Auxeticity of cubic and other crystalline solids can be found in some crystallographic directions^3^,^4^,^6^,^35^. About 70% cubic bulk materials show negative Poisson’s ratio along 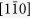-direction as they are stretched along [110]-direction^3^. We investigate behavior of Poisson’s ratio component along 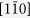-direction when they are stretched in [110]-direction of FCC Au by using MS simulation. We assigned *x*-, *y*-, and *z*-directions to be [110], 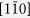, and [001]-directions, respectively. For [110] nanowires, the dimension *b* along the z-direction is kept as 17a_0_ while the dimension *a* along the *y*-direction can be 17a_0_ or smaller (9a_0_). We also considered the two more cases: First, when *a* becomes infinite and *b* is 17a_0_ so that the structure now becomes 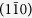 nanoplate; and, second, when *b* becomes infinite and *a* is 17a_0_ so that the structure now becomes (001) nanoplate. All structures are under uniaxial stress along [110]-direction. Details of the MS simulations can be found in the Simulation Detail section. In Fig. 7, we plot the change of the Poisson’s ratio component *ν*_*xy*_ of the nanostructures as well as that of the corresponding bulk material with strain. Milstein and Huang used analysis of elastic instability of cubic materials to explain the existence of the auxeticity^4^ in the cubic bulk materials. It is clear that *ν*_*xy*_ of all structures show auxeticity even at unstrained state. A negative Poisson’s ratio was observed experimentally in [110] nanowire^36^.

The mechanism for auxeticity of [100] structures is different from that of [110] structures. In the case of [110] structure, negative Poisson’s ratio is an *intrinsic* property regardless of structure size, whereas [100] structures show negative Poisson’s ratio behavior at nanoscale while they do not have auxeticity at bulk-scale. In addition, the role of surface stress in [100] nanostructures is more significant because the Poisson’s ratio can be tuned from positive at bulk-scale to negative at nanoscale, whereas surface stress in [110] nanostructure change slightly the Poisson’s ratio value. As discussed in the previous study^16^, this is a unique property of cubic materials as they are loaded along [100]-direction. Under uniaxial stress condition along [100]-direction, cubic material experiences elasticity and then fails with an elastic instability^37^. At the onset of elastic instability, sudden branching of crystal, a suddenly large contraction along a lateral direction and a suddenly large expansion along the other lateral direction, is observed^38^. At nanoscale, with the occurrence of the asymmetric induced stresses along the [010]- and [001]-directions, the sudden change branching is replaced by a smooth branching (Fig. 2a–c). Consequently, negative Poisson’s ratio can be observed in [100] nanostructures at sufficient strain. Because the branching of crystal is the unique property of cubic material under loading along [100]-direction, we do not observed the same phenomenon, i.e., smooth branching of the crystal in nanostructures with other crystallographic directions^39^,^40^.

**Figure 8:**
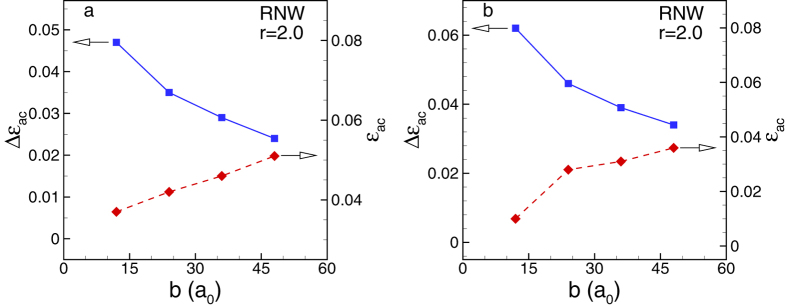
Size effect on auxeticity of nanostructures. (**a**) Au [100] RNW, and (**b**) Au [100] RNT. The aspect ratio of the cross-section of all structures is kept as 2.0. In the case of RNTs, we chose *c* = *b*, and *d* = *b/*6. When the size is smaller, both RNWs and RNTs are more auxetic i.e., the critical auxetic strain *ε*_*ac*_ is smaller whereas the auxetic strain range Δ*ε*_*ac*_ in elastic regime is larger.

We conducted more MS simulations to investigate the size effect on auxeticity of Au [100] RNWs and RNTs. The aspect ratio of all structures is fixed as 2.0. In the case of RNTs, we chose *c=b*, and *d=b/*6. In Fig. 8, we plot the change of the critical auxetic strain *ε*_*ac*_ and the auxetic strain range Δε_*ac*_ with the size. The auxetic strain range is defined as Δε_*ac*_=ε_*F*_ − ε_*ac*_ where ε_*F*_ is the failure strain of the structure. It is clear that the size strongly affects the Poisson’s ratio behavior of the nanostructures. As the size increases, the critical auxetic strains of both RNWs and RNTs increase whereas the auxetic strain ranges of the both nanostructures decrease. For example, when the cross-section is 24*a*_*0*_ × 12*a*_*0*_ (10 nm × 5 nm), the values of *ε*_*ac*_ and Δε_*ac*_ are 0.037 and 0.047, respectively. With the cross-section 96*a*_*0*_ × 48*a*_*0*_ (39 nm × 20 nm), the RNW shows negative Poisson’s ratio at relatively large critical auxetic strain of about 0.05 whereas the auxetic strain range is relatively small 0.024.

We note that one would obtain different values of the critical auxetic strain and auxetic strain range if the nanostructures are considered at higher temperature. For example, in Figure S4, we compared the change of the strain along the *z*-direction of a RNT with the applied strain at different temperatures. The results were obtained by using molecular dynamics (MD) simulation. Details on MD simulations can be seen in the Simulation Methods section. Clearly, the RNT is more auxetic at higher temperature due to the change of elastic moduli with temperature^18^. However, as can also be seen in Figure S4, the RNT at higher temperature fails at earlier strain because the nucleation stress is smaller^41^. In addition, the sharp corners in the geometry can lead to early failure especially at high temperature^41^. The yield strain of sharp corner nanowire e.g., square nanowire is much smaller than that of round corner nanowire e.g., circle nanowire^42^. We can avoid the early failure so that auxetic behavior can be observed in larger strain range by considering rectangular structures with round corners or other asymmetric cross-section shapes such as ellipse. We confirmed that auxetic behavior of ellipse NW and NT are similar to those of RNW and RNT (Figure S5).

It is important to mention that the nanostructures in reality might fail before they can show auxetic behavior even if round-shape corners are introduced as mentioned above. This is because pre-existing defects such as dislocation, grain boundary etc. can move at a strain smaller than a critical auxetic strain. However, at nanoscale, low defect density structures or defect-free structures^18^,^43^,^44^,^45^ can be synthesized. It was reported in experimental studies that elastic strains of defect-free nanostructures become much larger than those of the corresponding bulk materials e.g., 0.072 for Cu nanowires^18^. Therefore, while negative Poisson’s ratio might be hard to be observed in high defect density structures, we believe that it is highly possible to observe the phenomenon in defect-free or low defect density nanostructures in reality. We hope that future experimental works can provide clear evidence that negative Poisson’s ratio can be observed in the RNWs and RNTs.

## Conclusions

In summary, we have shown that positive Poisson’s ratio of the FCC metals can be turned into negative at finite strain if an asymmetric cross-section of nanowires such as rectangle or ellipse is introduced. The degree of the asymmetry of the induced compressive stresses by surface relaxation at nanoscale metals is a main origin of the auxetic behavior of the metal (001) nanowires. In addition, we have shown that by introducing a hole inside the nanowires, the effect of surface can become more profound so that the auxeticity can be significantly improved. We provide a new design method in which dimensions of the hole is controlled for tuning the Poisson’s ratio to the desired value. Finally, we have shown that the Poisson’s ratio of the one-dimensional nanoscale structures can be effectively controlled by changing the base metal. Metals with larger surface stress exhibit more auxetic behavior at the same geometric condition at nanoscale, although the metals have almost the same Poisson’s ratio at bulk-scale. This work contributes to the library of auxetic materials at nanoscale with a distinct mechanism.

### Simulation methods

We mainly employed MS simulation to predict response of nanoplates, nanowires and nanotubes under loading using Large-scale Atomic/Molecular Massively Parallel Simulator (LAMMPS)^46^. We modeled the FCC [100] nanostructures assigning the [100]-, [010]-, and [001]-directions to the *x*-, *y*-, and *z*-directions, respectively. FCC [110] nanostructures were utilized assigning the [110]-, 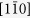-, and [001]-directions to the *x*-, *y*-, and *z*-directions, respectively. To model nanoplates, we assigned periodic boundary condition (PBC) along the *x*- and *y*- (or *z*-) directions while a large vacuum was created in the *z*- (or *y*-) direction to make free surface. To model infinite nanowires and nanotubes, PBC is imposed along the *x*-direction while large vacuum was created in the *y*- and *z*-directions. Conjugate gradient method was used for all minimization processes in MS simulations. In order to save computational cost and avoid some possible divergence of minimizations, the periodic length of models was chosen 4*a*_*0*_. 4*a*_*0*_ is enough because the mechanical quantity of the metals was investigated within their elastic regime only. To underline the generality of our finding, the interactions between the atoms of the nanoscale metals are described by different embedded-atom method (EAM) potential models, which were developed by Foiles *et al*^27^, Cai and Ye^47^, Liu *et al*^48^. In the MS calculations, we minimized the total energy of the system and obtained the stable state corresponding to force equilibrium under the given loading conditions.

Before loading is applied, each system is relaxed to get equilibrium state. Then, we stretch the nanoscale materials with an incremental true strain of 0.001 along the *x*-direction. To simulate uniaxial stress condition (*σ*_*x*_ ≠ 0, others zero), periodic box of nanoplates is adjusted along the *y*-direction of nanoplates to satisfy the stress free condition.

The Poisson’s ratio along the *x*-direction for a material under loading is defined as




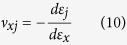




where the subscript *j* can be *y* (in-plane lateral direction) or *z* (thickness direction). We used the central difference method with second-order accuracy to obtain the first derivative of the lateral strain in Equation (10). Therefore, we could obtain the value of the Poisson’s ratio at a strain 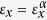 as follows:




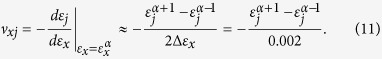




We also employed MD simulations using LAMMPS to investigate the effect of temperature on auxetic behavior of nanostructures. We first relaxed the system to obtain the equilibrium state by using MS simulation, and then increased temperature of the system from 0 K to 600 K using Langevin dynamics over 100 ps. We annealed the system at 600 K over 50 ps under NPT ensemble in which the stress along the *x*-direction becomes zero. We applied strain along the *x*-direction with the strain rate of 10^9^ s^−1^.

## Additional Information

**How to cite this article**: Ho, D. T. *et al* Metal [100] Nanowires with Negative Poisson's Ratio. *Sci. Rep.* **6**, 27560; doi: 10.1038/srep27560 (2016).

## Supplementary Material

Supplementary Information

## Figures and Tables

**Table 1:** Comparison of mechanical properties of FCC cubic RNWs at the unstrained state.

Metal	*ν* (bulk)	*ν*_*xy*_(RNT)	*ν*_*xz*_(RNT)	*ε*_*ac*_(RNT)	*f*(J/m^2^)
Pt	0.47	0.67	0.06	0.007	2.647
Pd	0.46	0.61	0.16	0.017	2.000
Au	0.46	0.61	0.17	0.018	1.572
Cu	0.42	0.54	0.21	0.024	1.396
Ni	0.40	0.48	0.24	0.030	1.320
Ag	0.41	0.48	0.27	0.036	0.815

*ε*_*ac*_ is the critical strain at which the structure become auxetic; *f* is the surface stress along the [100]-direction in (100) crystal face.
