# Supplementary material for: Metal [100] Nanowires with Negative Poisson’s Ratio
Source: Sci Rep. 2016 Jun 10;6:27560. doi: 10.1038/srep27560 (PMC4901344; doi:10.1038/srep27560)
Supplement: Supplementary Information [file srep27560-s1.pdf]

Supplementary information:

Metal [100] Nanowires with Negative Poisson's Ratio

Duc Tam Ho[1], Soon-Yong Kown[2], and Sung Youb Kim[1*]

[1]Department of Mechanical Engineering, Ulsan National Institute of Science and Technology, Ulsan 44919, South Korea.

[2]School of Materials Science and Engineering, Ulsan National Institute of Science and Technology, 44919, South Korea

(2016.05.08)

[*]To whom correspondence should be addressed. e-mail: sykim@unist.ac.kr

Stress fields in rectangular nanowires

In Figure S1, we compare the stress fields of Au nanoplate, SNW, and RNW. The aspect ratios of the nanoplate, SNW, and RNW are ∞, 1.0, and 2.0, respectively, and all the nanoscale structures have the same thickness of $50a_0$. In the case of the nanoplate, the stress filed σ_y is tensile on and near the surface and it is compressive and constant at the interior part. For the SNW, the stress fields σ_y and σ_z are similar owing to the symmetry of the cross-section and they are compressive and nearly homogeneous at the interior part. However, it is clear for the RNW that the stress field σ_z is much complicate and even tensile at the interior part, although the stress field σ_y is similar to that of the SNW, as shown in Figure S1. This complicate distribution of stress field σ_z at the interior part of the RNW originates from the different sizes of free surfaces of the RNW. Larger size of horizontal surfaces than vertical surfaces induces larger amount of compressive stress (as well as compressive deformation) in the *y*-direction, and this large compressive stress generates relatively tensile deformation in the *z*-direction owing to the Poisson's ratio effect. Therefore, in the *z*-direction, the relatively

tensile deformation induced by the horizontal surfaces compensates the compressive deformation induced by the tensile stress on the vertical surfaces. Through this interaction, which is the correlation of the relaxations of the horizontal and vertical surfaces of the RNW, there occurs a complicate and inhomogeneous distribution of stress field σ_z at the interior part. This is an intrinsic mechanical characteristic of metal RNWs

We further investigate this inhomogeneous distribution. In Figure S2, the stress distribution σ_z of RNWs with different aspect ratios are compared. Here, *a* is various with the aspect ratio *r* ranging from 1.5 to 5.0, while *b* is kept as $b = 50a_0$. As shown in Figure S2, the distribution of the stress field σ_z of the RNWs depends strongly on their aspect ratios. When the aspect ratio is 1.6, a tensile stress zone (TSZ) begins to occur in the interior part of the RNWs, and it is split into two separate TSZs as the aspect ratio becomes sufficiently large (>3.0). The separate TSZs are always positioned at certain places: the distance from the center of TSZs to the nearest side of the RNWs is approximately the same with the thickness of the RNWs. It is worth noting that even though the average stresses $\bar{\sigma}_z$ of the RNWs are always compressive, the TSZs can exist. Interestingly, two separated TSZs are clearly observed when the aspect ratio of the RNW is sufficiently large (> 3.0) and the two TSZs are overlapped with each other as the aspect ratio is smaller.

Based on the observation, a question may arise: Why is a TSZ not seen in RNWs of which aspect ratio smaller than 1.6? Our answer to the question is in the following. Our simulations show that the distance from the boundary of a TSZ to the nearest side surface of all RNWs in Figure S2 is approximately 0.8*b*. It indicates that once the aspect ratio is smaller than 1.6, the required distance 0.8*b* for a TSZ is not satisfied. This is why a RNW with the aspect ratio smaller than 1.6 does not have a TSZ. Remarkably, again, the behavior of the stress field σ_z and the TSZs are the same for other RNWs with different sizes (Figure S3). In addition, we

confirm that these behaviors can be observed in other metals and other potential models (Refs. 47 and 48 in the main text).

The TSZs increase the degree of asymmetry of the induced stresses in the interior part of RNWs, and thus the auxeticity of the RNWs can be significantly enhanced as presented in the main text of this report. The existence of TSZ(s) is the unique property of the RNWs with the sufficiently large aspect ratio.

Poisson's ratio in [100] ellipse nanowires and nanotubes

We have shown in the main text that RNWs and RNTs can exhibit negative Poisson's ratios. We believe that the negative Poisson's ratio can be found not only in RNWs and RNTs but also in any metal nanowires or nanotubes with any polygonal as well as elliptic cross-section which is asymmetric from the y-direction to the z-direction. The asymmetry of cross-section provides different amounts of the induced stresses in the lateral directions and thus makes auxetic behavior of the nanowires (or nanotubes) possible. The induced stresses are generated by the surface relaxation, and the asymmetry of them comes from the geometric asymmetry of cross-sections. Therefore, the auxetic behavior is an intrinsic property of nanoscale metal nanowires and nanotubes of which cross-section is asymmetric. Figure S5 shows Poisson's ratios of the Au [100] elliptical nanowire with major radius of $a=18a_0$ and minor radius of $b=9a_0$, and the corresponding Au [100] elliptical nanotube with the same a, b, and the thickness of $t=6a_0$. Auxeticity is observed in both nanowire and nanotube. Similar to the case of rectangular cross-section, elliptical nanotube is more auxetic than elliptical nanowire.

Figures

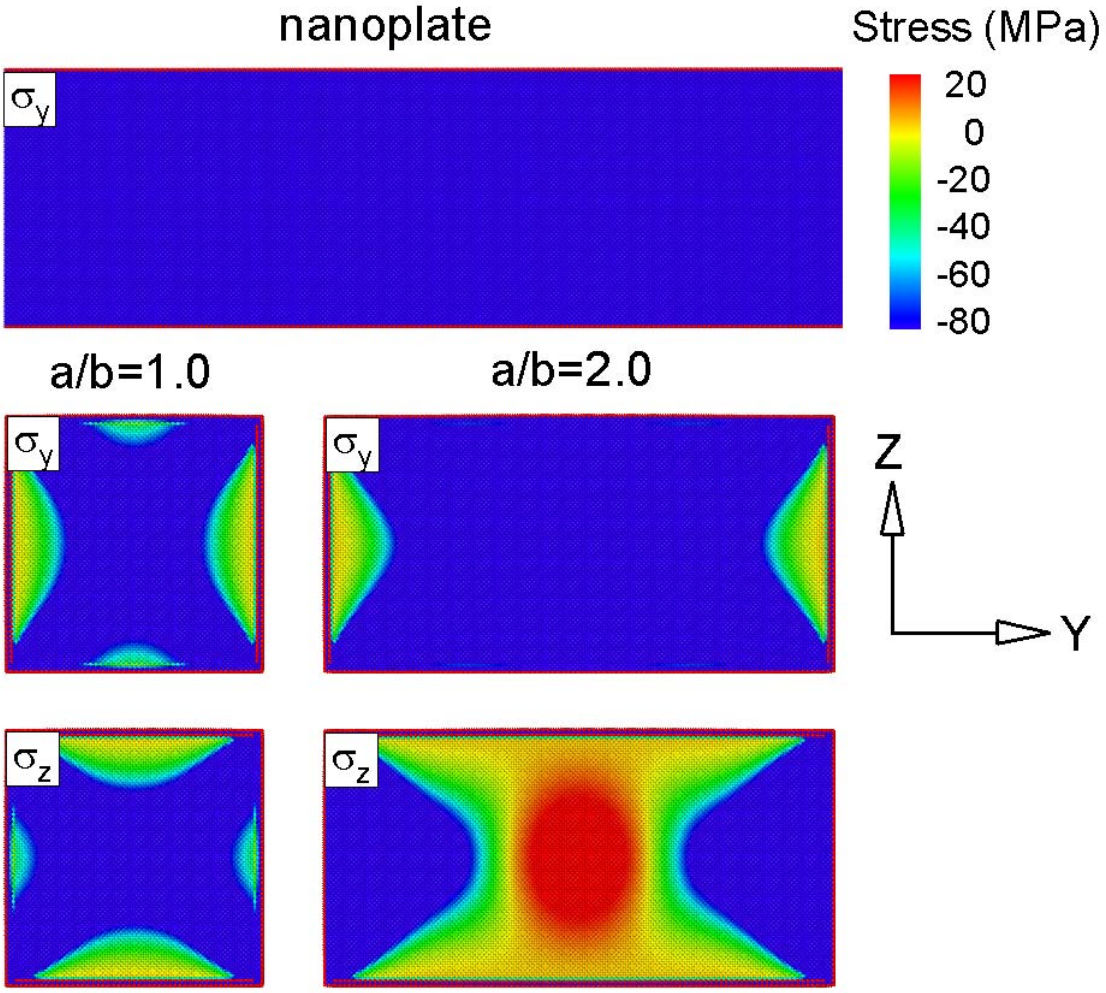

Figure S1: Stress fields in the cross-sections of a SNW, RNW, and nanoplate. All of the nano-structures have the same thickness b=50a_0. The distribution behaviors of the stresses in the interior parts of all structures are homogeneous and compressive (blue), except σ_z of the RNW (red). σ_z of the RNW is inhomogeneous and even becomes tensile when the aspect ratio is sufficiently large.

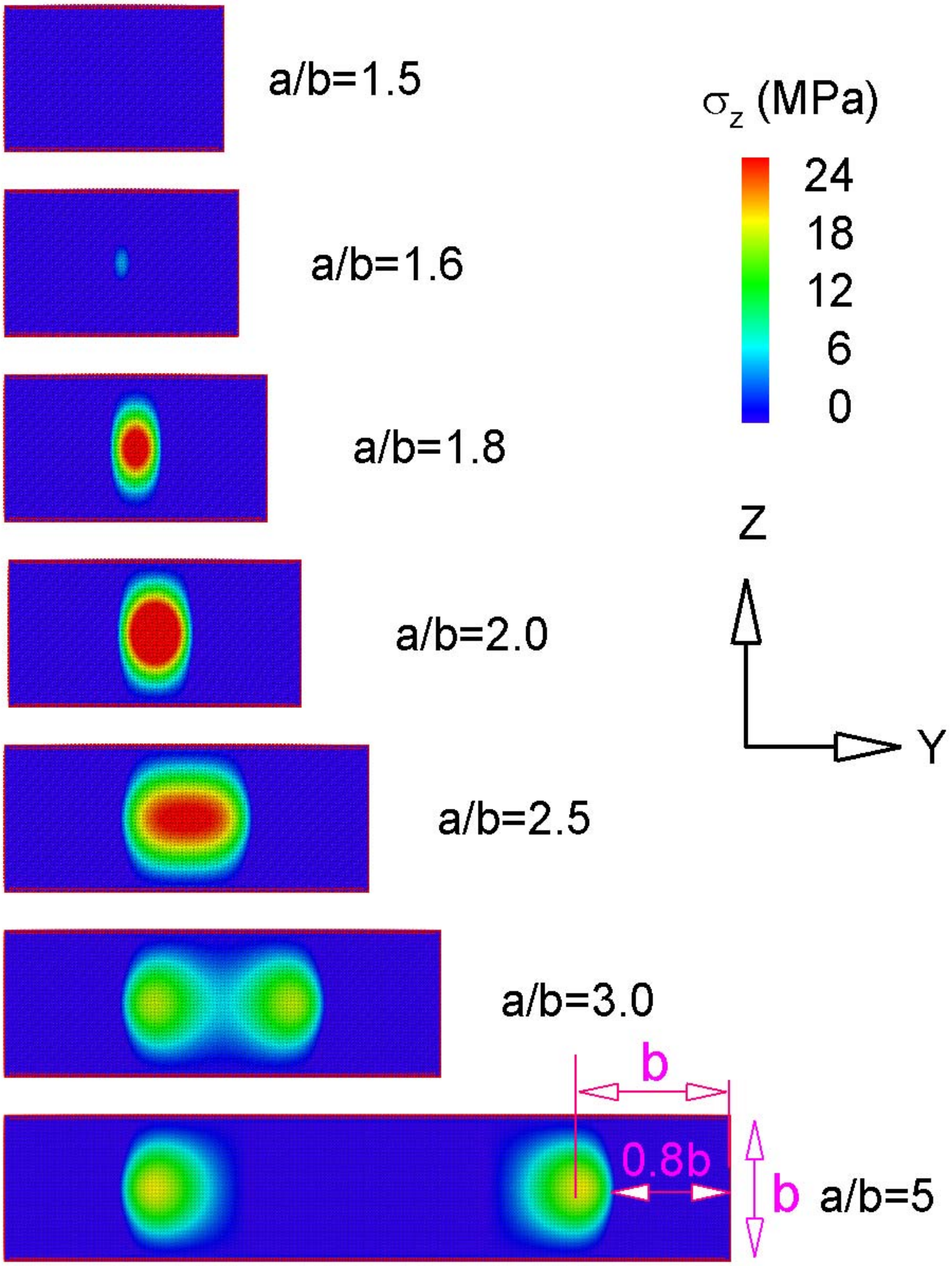

Figure S2: Stress fields σ_z in the cross-sections of RNWs with different aspect ratios. All of the structures have the same thickness b=50a$_0$. The stress distributions of the σ_z are strongly dependent on the aspect ratio. When the aspect ratio is larger than 1.5, a TSZ takes place, and it is split into two separate TSZs as the aspect ratio becomes sufficiently large (>3.0). The distance from the nearest side to the center of the TSZ is approximately the same

with the thickness of the RNW, and the distance to the closest boundary of the TSZ is approximately 80% of the thickness.

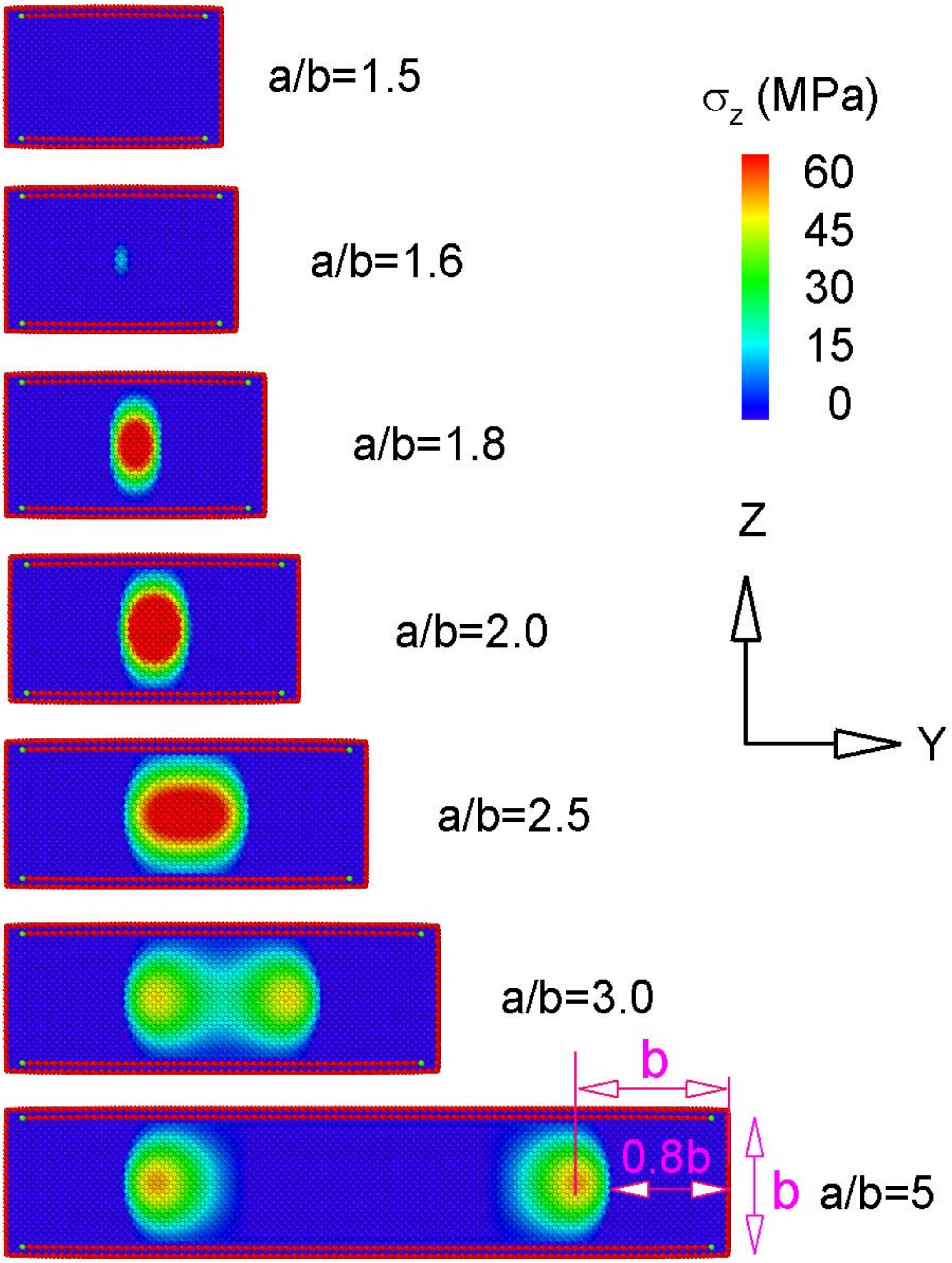

Figure S3: Stress fields σ_z in cross-section of thinner RNWs with different aspect ratios. All of the structures have the same thickness b=18a$_0$. Remarkably, the behavior of the stress field is the same as that of the RNWs shown in Figure S2.

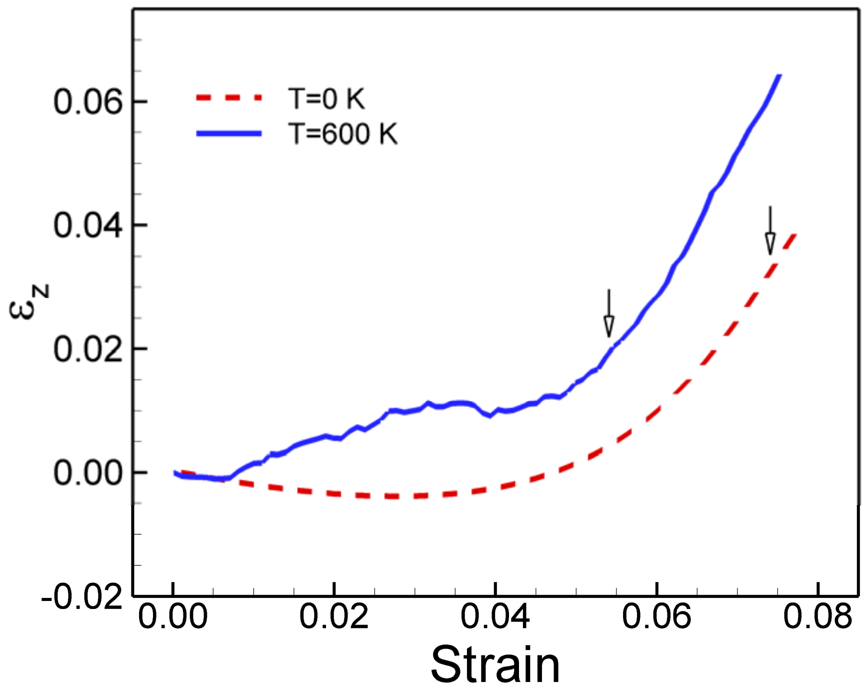

Figure S4: Mechanical response of an Au [100] RNT at different temperatures. The dimensions of the RNT are: a=48a_0, b=24a_0, c=24a_0, and d=4a_0. The RNT becomes more auxetic at higher temperature but it fails at smaller strain. The arrows present the onset of failure of the structures.

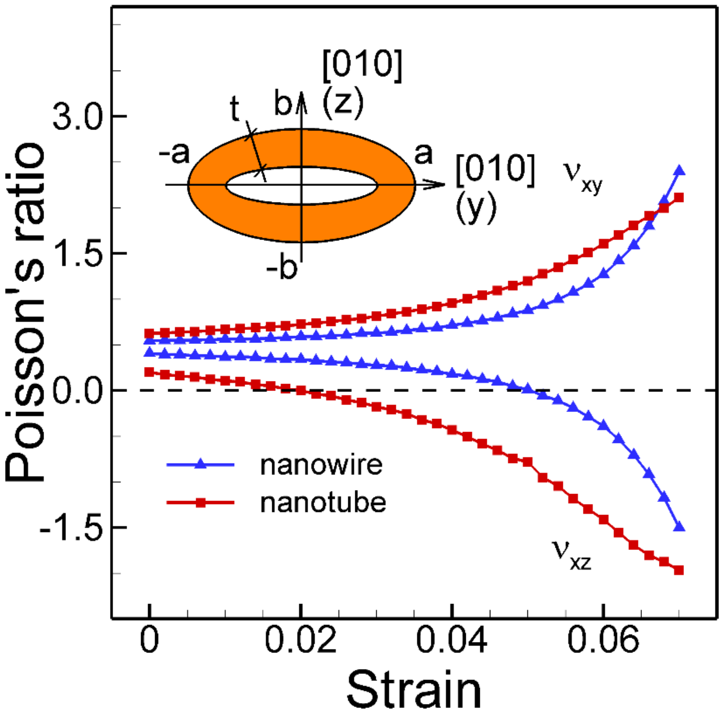

Figure S5: Poisson's ratios of an Au (001) elliptical nanowire and an Au (001) elliptical nanotube. For the nanowire, the major radius a=18a₀, and minor radius a=9a₀. The nanotube has the same parameters a, b of the nanowire, and the thickness of the nanotube is t=6a₀. Both exhibit auxeticity because of the asymmetry of the cross-sections, and similar to the case of RNWs, the elliptical nanotube is more auxetic than the elliptical nanowire.
